# Deep learning image reconstruction technique for improving image quality and radiation dose reduction compared to iterative reconstruction technique in non-contrast CT head imaging

**DOI:** 10.3389/fradi.2026.1824489

**Published:** 2026-06-16

**Authors:** Obhuli Chandran M., Saikiran Pendem, Rajagopal Kadavigere, Priyanka, Priya PS, Cijo Chacko

**Affiliations:** 1 Department of Medical Imaging Technology, Manipal College of Health Professions, Manipal Academy of Higher Education, Manipal, India; 2 Department of Radiodiagnosis and Imaging, Kasturba Medical College, Manipal Academy of Higher Education, Manipal, India; 3 Philips Research and Development, Philips Innovation Campus, Yelahanka, Karnataka, India

**Keywords:** contrast-to-noise ratio, deep learning image reconstruction, image noise, iterative reconstruction, non-contrast computer tomography (NCCT), signal-to-noise (*S*/*N*) ratio

## Abstract

**Introduction:**

Non-contrast CT (NCCT) head is a standard imaging modality for evaluating central nervous system pathologies. Repeated CT brain examinations are associated with a cumulative risk of cancer development. Deep learning image reconstruction (DLIR) techniques help improve image quality (IQ) while reducing radiation dose (RD) compared with iterative reconstruction techniques (iDose^4^). Therefore, our study compared IQ parameters among low-dose (LD) CT with DLIR (Precise Image), LD CT with iterative reconstruction (iDose^4^), and standard-dose (SD) CT with iDose^4^ and further evaluated radiation dose reduction between SD-iDose^4^ and LD-DLIR in NCCT head imaging.

**Methods:**

Group A (SD with iDose^4^) and Group B (LD with DLIR; Precise Image) each included 96 patients. All NCCT brain scans were performed using a 128-slice incisive CT scanner (Philips Healthcare Systems) with iDose^4^ and DLIR. Qualitative and quantitative IQ analyses and radiation dose indices were compared between the groups. Lesion conspicuity was also assessed between LD-DLIR and LD-iDose^4^.

**Results:**

There was a significant reduction in RD (effective dose: 1.01 vs. 2.4 mSv; *p*<0.05) with LD-DLIR. LD-DLIR demonstrated superior IQ compared with both SD-iDose^4^ and LD-iDose^4^. Subjectively, gray–white matter differentiation improved from scores of 3 (SD-iDose^4^) and 2.5 (LD-iDose^4^) to 4.5 (LD-DLIR), while overall image quality and subjective image noise increased from 3–3.5 (SD-iDose^4^) and 2.5–3 (LD-iDose^4^) to 4–4.5 with LD-DLIR (*p* < 0.05). Image noise was lowest with LD-DLIR in gray matter thalamus (GMT: 2.30–2.31) compared with SD-iDose^4^ (4.80–4.85) and LD-iDose^4^ (6.15–6.25); similar results were seen in the white matter posterior limb of the internal capsule (WMPIC: 1.98–2.21 vs. 4.36–4.38 vs. 5.19–5.32) and adjacent cortical gray matter (ACGM: 2.17–2.19 vs. 4.14–4.22 vs. 5.04–5.08). SNR was highest with LD-DLIR [GMT: 16.29–16.36 vs. 7.32–7.34 (SD-iDose^4^) and 5.61–5.75 (LD-iDose^4^); WMPIC: 14.69–14.75 vs. 6.49–6.51 vs. 5.18–5.26], and contrast-to-noise ratio (CNR) was also markedly improved [GMT–WMPIC: 2.59–2.61 (LD-DLIR) vs. 0.98–0.99 (SD-iDose^4^) and 0.82–0.83 (LD-iDose^4^); ACGM–FWM: 2.15–2.29 vs. 0.93–0.95 vs. 0.85–0.86]. Lesion conspicuity was superior with LD-DLIR for all brain lesions compared with LD-iDose^4^.

**Conclusion:**

Our study demonstrated that LD NCCT head imaging with DLIR provides superior noise reduction and improved GWMD, SNR, and CNR compared with SD and LD with iDose^4^ and supports the clinical utilization of DLIR.

## 1 Introduction

Non-contrast computed tomography (NCCT) head remains the frontline imaging modality for evaluating central nervous system pathologies because of its faster image acquisition, high spatial resolution, widespread availability, and cost-effectiveness (1–3). CT scans increase the risk of cancer due to greater radiation exposure (4). Repeated CT brain scanning can increase the cumulative risk of cancer development. Since head CT scans are commonly conducted and often repeated in the same patients, it is important to implement dose optimization techniques in clinical practice to minimize the potential adverse effects associated with cumulative exposure to ionizing radiation (5–7). CT brain imaging is quite challenging because the brain is surrounded by bones and suffers from photon starvation, leading to beam hardening and partial-volume artifacts in the cranial fossa (8).

For more than three decades, filtered back projection (FBP) has been the traditional reconstruction method because of its computational efficiency and accuracy. However, at lower radiation doses, it produces noisy images and artifacts (9). Consequently, iterative reconstruction (IR) algorithms have addressed this limitation by reducing noise and improving image quality (IQ) at lower radiation levels with reduced peak kilovoltage (kVp) and tube current × exposure time (mAs). iDose^4^, developed by Philips Healthcare (Cleveland, OH, USA), is a fourth-generation iterative reconstruction technique designed to enhance image quality while implementing dose-reduction strategies (10, 11). However, IR techniques at higher reconstruction strengths result in a plastic or blotchy appearance (12–14).

Deep learning image reconstruction (DLIR) techniques have been introduced to improve IQ and possibly reduce radiation dose (RD). DLIR algorithms have the potential to surpass current reconstruction methods by effectively reducing image noise and artifacts (15–17). Precise Image is an advanced CT image reconstruction algorithm developed by Philips Healthcare that uses deep learning methods to deliver high-quality images at significantly lower radiation doses, with reduced kVp and mAs, while maintaining a traditional filtered back projection-like appearance. The algorithm is trained using a convolutional neural network to reconstruct low-dose scan data to match routine-dose FBP-quality images, effectively maintaining spatial resolution and natural noise texture without a plastic-like look often associated with IR methods. Clinical evaluations and quality assurance measures, such as “noise power spectrum (NPS), modulation transfer function, and detectability index (d-prime),” demonstrate that Precise Image enables up to 80% better low-contrast detectability at lower doses and achieves faster reconstruction times typically under 30 s (18).

Radiation dose in CT increases nearly with the square of the tube voltage while maintaining a linear dependence on mAs. Therefore, when both kVp and mAs are reduced simultaneously, dose reduction becomes multiplicative: fewer photons are produced (mAs effect), and each photon has lower energy (kVp), which results in a substantial overall decrease in patient radiation exposure (19, 20). Excessive reduction of both parameters may increase image noise (IN) and reduce image quality, which can be mitigated using advanced reconstruction techniques such as DLIR. To the best of our knowledge and literature search, there are no studies evaluating subjective and objective IQ and RD using a low-dose (LD) protocol with reduction of kVp and mAs simultaneously for adult NCCT head examinations using DLIR (Precise Image). Therefore, we compared the IQ parameters among low dose (LD) with DLIR (Precise Image), LD with iterative reconstruction (iDose^4^), and standard dose (SD) with iDose^4^ and further evaluated radiation dose reduction between SD-iDose^4^ and LD-DLIR in NCCT head imaging.

## 2 Materials and methods

This is a prospective study. The study was approved by the Institutional Ethics Committee of our hospital. Written informed consent was obtained from each patient before the scan.

An initial phantom study was conducted using an adult head CT polymethyl methacrylate (PMMA) phantom. IN was analyzed and quantified between the SD and LD settings on the phantom.

Then, a human study was performed in two phases. The sample size was calculated based on the comparison of means between two independent groups using the following formula:

$$n=(2\sigma^{2}{\left(Z_{1-\alpha /2}+Z_{1-\beta }\right)}^{2})/d^{2}$$

A two-sided significance level (*α*) of 0.05 and a statistical power of 80% (*β* = 0.20) were assumed, corresponding to *Z* values of 1.96 and 0.84, respectively. The standard deviation (*σ*) was 1.9, and the difference (*d*) between the groups was set at 0.77.

Phase I consisted of Group A, which included 96 patients who underwent the SD NCCT head protocol (120 kVp, 290 mAs, iDose^4^; level 3). Phase II consisted of Group B, which included 96 patients who underwent the LD NCCT Head protocol (100 kVp, 200 mAs, DLIR-standard; Precise Image). The LD images of Phase II were reconstructed using DLIR (Standard) and IR (iDose^4^; level 3). Indications for adult NCCT head examinations included intracranial hemorrhage, infarction, small vessel ischemic changes, cerebral atrophy, tumor, and postoperative cases.

### 2.1 Phantom study

An adult head CT PMMA phantom was scanned using low-dose parameters of 100 kVp with mAs values of 100, 150, and 200 reconstructed with DLIR, and standard-dose parameters of 120 kVp and 290 mAs reconstructed with iDose^4^ to determine the optimum low-dose parameters. We measured IN by drawing a region of interest (ROI) of 0.3 cm^2^ for both groups.

### 2.2 Human study

#### 2.2.1 Image acquisition

Patients referred for NCCT head examination underwent scanning on a Philips CT scanner (128-slice Incisive CT, Philips Healthcare, Cleveland, OH, USA). The NCCT brain scanning parameters are provided in Table 1.

**Table 1:** Detailed acquisition parameters for groups A and B on the 128-slice Philips Incisive CT scanner.

Parameters	Group A (standard dose)	Group B (low dose)
Tube voltage	120 kVp	100 kVp
Tube current	290 mAs	200 mAs
Rotation time	0.5 s	0.5 s
Helical pitch	0.70	0.70
Collimation	32 × 0.625	32 × 0.625
Slice thickness	3	3
FOV	250	250
Image matrix size	512 × 512	512 × 512
Reconstruction algorithms	iDose^4^, level 3	Precise image DLIR-standard iDose^4^, level 3

#### 2.2.2 Subjective image quality

**Figure 1:**
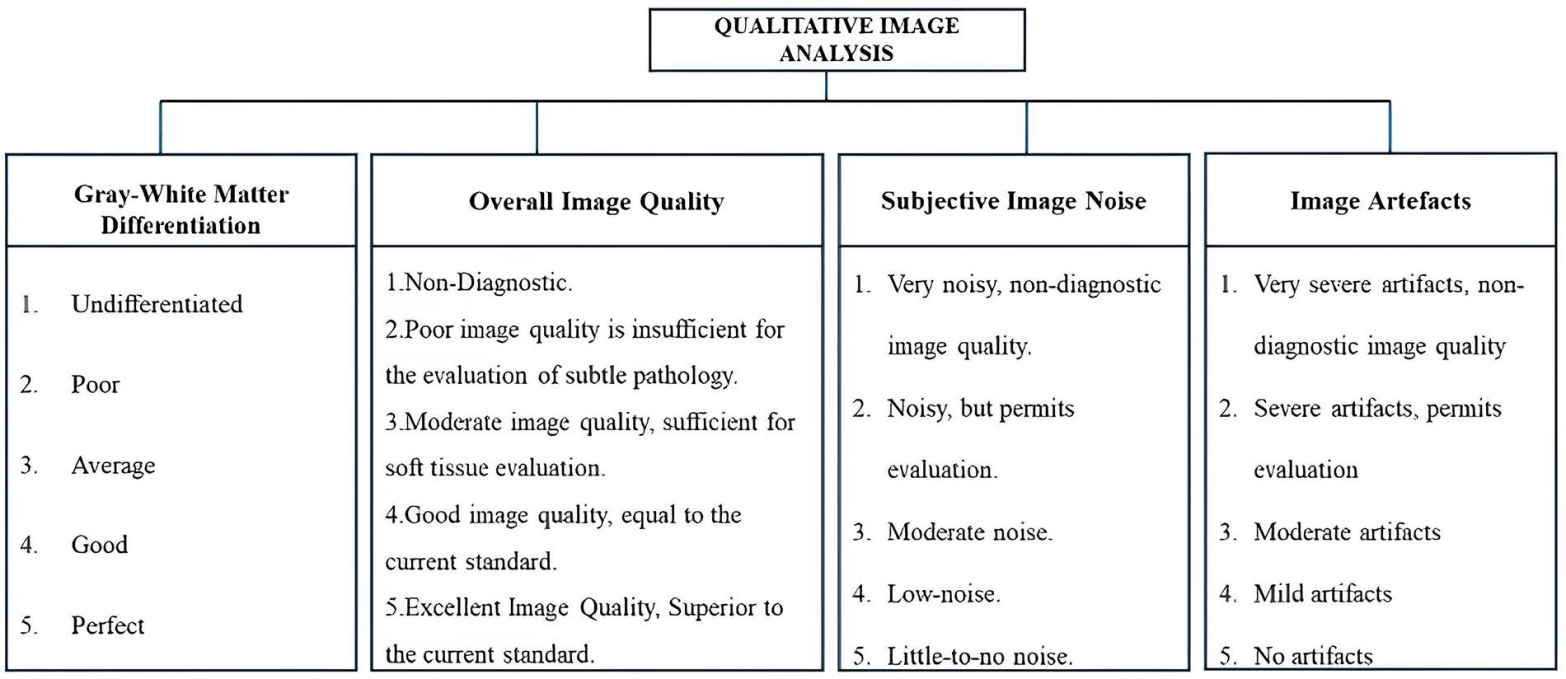
Parameters used to assess the qualitative image analysis

Two radiologists, each with over 10 years of experience, independently evaluated CT brain images reconstructed using SD- iDose^4^, LD- iDose^4^, and LD-DLIR techniques. The radiologists were blinded to the NCCT head protocols and reconstruction methods. Gray–white matter differentiation (GWD), overall image quality (OIQ), subjective image noise (SN), and image artifacts (IA) were evaluated using a five-point Likert scale (20), as shown in Figure 1.

#### 2.2.3 Objective image quality

**Figure 2:**
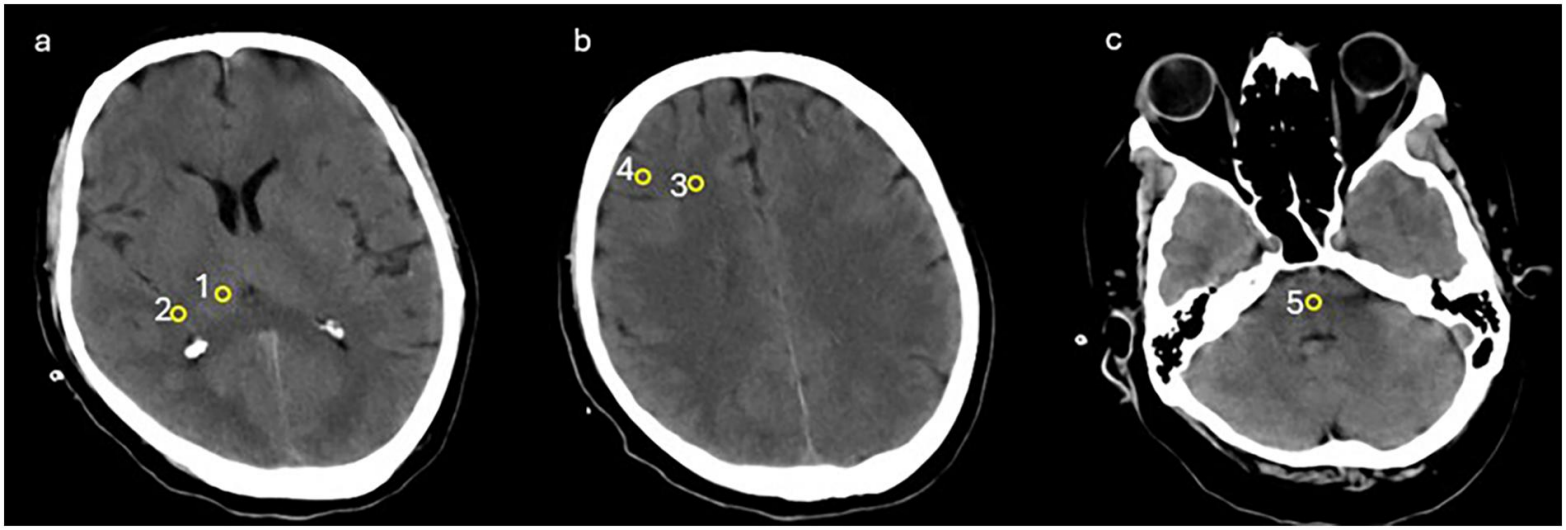
Axial CT image of a 65-year-old man showing the region of interest at the **(a)** basal ganglia level (BGL): 1—gray matter thalamus (GMT), 2—white matter posterior limb of the internal capsule (WMPIC); **(b)** centrum semiovale level (CSL): 3—adjacent cortical gray matter (ACGM), 4—frontal white matter; and **(c)** 5—pons.

IQ parameters, such as IN, signal-to-noise ratio (SNR), and contrast-to-noise ratio (CNR), were measured at the basal ganglia level (BGL) and centrum semiovale level (CSL) for the NCCT head images reconstructed using SD-iDose^4^, LD-iDose^4^, and LD-DLIR techniques. IN was defined as standard noise in the ROI placed in the gray matter thalamus (GMT) and white matter posterior limb of the internal capsule (WMPIC) at the BGL and in the adjacent cortical gray matter (ACGM) and frontal white matter (FWM) at the CSL (Figures 2a–c). The signal-to-noise ratio (SNR) at the BGL and CSL was measured as CT attenuation [Hounsfield unit (HU)]/IN.

The CNR at the BGL and CSL was measured using the following formula:

$$\begin{array}{rl} \mathrm{CNR} & =(\mathrm{Mean}\;HU_{\mathrm{GMT}/\mathrm{ACGM}}-\mathrm{Mean}\;HU_{\mathrm{WMPIC}/\mathrm{FWM}})/\\ & \;\surd ({\left(SD_{\mathrm{GMT}/\mathrm{ACGM}}\right)}^{2}+\;{\left(SD_{\mathrm{WMPIC}/\mathrm{FWM}}\right)}^{2} \end{array}$$

The posterior fossa index (PF) was measured as IN, defined by the standard deviation (SD) of HU within the pons. The region of interest measuring 0.1–0.2 cm^2^ was placed in the GMT, WMPIC at BGL, FWM, ACGM at the CSL, and in the pons region within the posterior cranial fossa.

#### 2.2.4 Radiation dose estimation

The volume CT dose index (CTDIvol), dose–length product (DLP), size-specific dose estimate (SSDE), and effective dose (ED) were recorded. The ED was calculated by multiplying the DLP by a conversion factor (K) of 0.0021 (21).

#### 2.2.5 Lesion conspicuity

Two radiologists, blinded to acquisition parameters, independently evaluated the conspicuity of intracranial lesions on LD-IR and LD-DLIR images. The radiologists rated the lesion conspicuity of each image (1, least conspicuous among two series; 2, most conspicuous among two series) based on lesion border sharpness and contrast relative to the adjacent surrounding structures. The mean and percentage scores of 1 and 2 from both readers were assessed.

### 2.3 Statistical analysis

**Figure 3:**
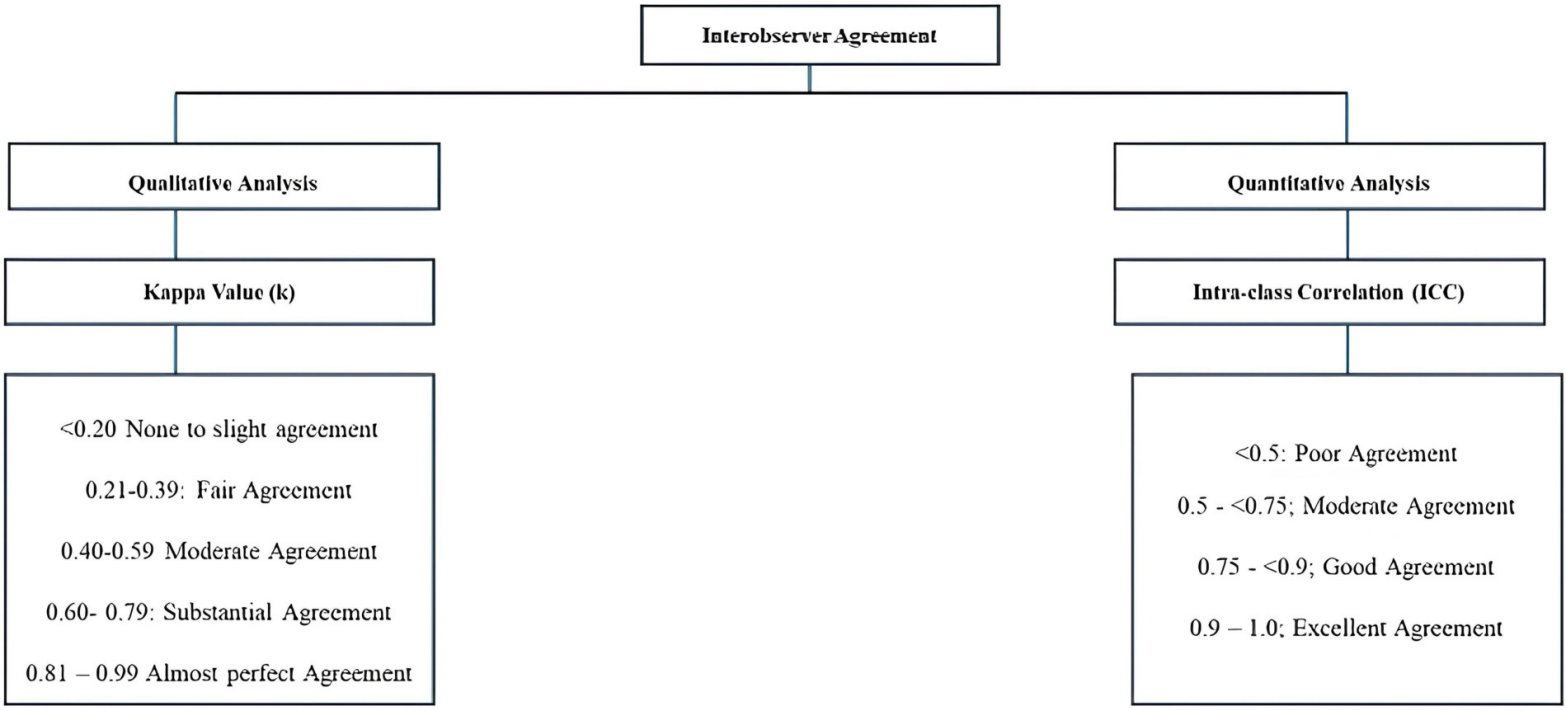
Cohen's kappa and intraclass coefficient (ICC) measures.

Statistical analysis was performed using Jamovi (version 2.3.26). Subjective and objective IQ analyses between SD-iDose^4^ vs. LD-iDose ^4^ and SD-iDose^4^ vs. LD-DLIR protocols were compared using the Mann–Whitney *U*-test. Subjective and objective IQ analyses for LD-iDose^4^ and LD-DLIR were performed using the Wilcoxon signed-rank test. An independent *t*-test was used to assess differences in RD between SD-iDose^4^ and LD-DLIR. The level of interobserver agreement for subjective IQ analysis was evaluated using Cohen's kappa coefficient, while interobserver agreement for objective IQ analysis was assessed using the intraclass correlation coefficient (ICC). The ranges for ICC and Kappa value are shown in Figure 3. Percentage scores of 1 and 2 for lesion conspicuity were assessed for the LD-iDose^4^ and LD-DLIR groups. A paired t-test was performed to compare lesion conspicuity scores between LD-iDose^4^ and LD-DLIR groups. *p* < 0.05 was considered statistical significance.

## 3 Results

A total of 192 patients were enrolled in the study. A total of 96 patients underwent the SD (120 kVp, 290 mAs) and LD (100 kVp, 200 mAs) NCCT head protocols. The demographic details are presented in Table 2.

**Table 2:** Demographic characteristics of groups A and B.

Demographic parameters	Group A	Group B
Male (*n*)	70	60
Female (*n*)	26	36
Age (mean ± SD)	52.5 ± 16.2	50.9 ± 19.2

### 3.1 Phantom study

**Figure 4:**
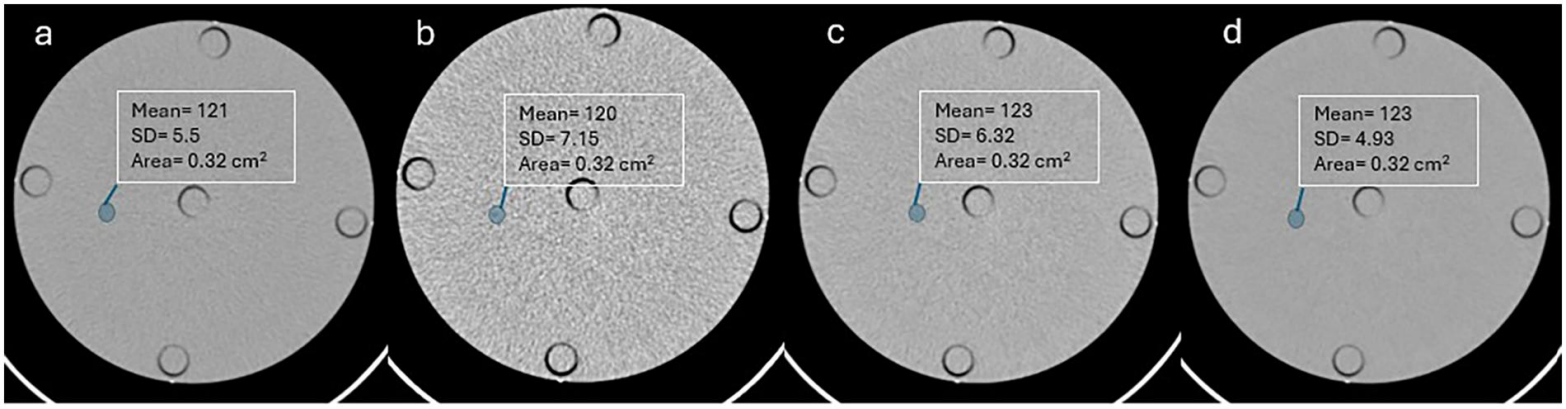
Image noise in the phantom with different exposure factors: **(a)** 120 kVp, 290 mAs; **(b)** 100 kVp, 100 mAs; **(c)** 100 kVp, 150 mAs; and **(d)** 100 kVp, 200 mAs.

The IN for the SD (120 kVp, 290 mAs; iDose^4^) group was 5.5. The image noise values for the LD protocols at 100 kVp with 100, 150, and 200 mAs were 7.15, 6.32, and 4.9, respectively (Table 3; Figures 4a–d). Our study noted that DLIR protocol using100 kVp and 200 mAs produced IN values closer to SD-iDose^4^. Therefore, 100 kVp and 200 mAs were chosen as the LD protocol and validated in the human study.

**Table 3:** IN comparison between SD and LD parameters in the phantom study.

IQ parameter	SD-120 kVp (iDose^4^)	LD-100 kVp (DLIR)		
	290 mAs	100 mAs	150 mAs	200 mAs
IN	5.5	7.15	6.32	4.9

### 3.2 Human study

#### 3.2.1 Subjective IQ analysis

**Figure 5:**
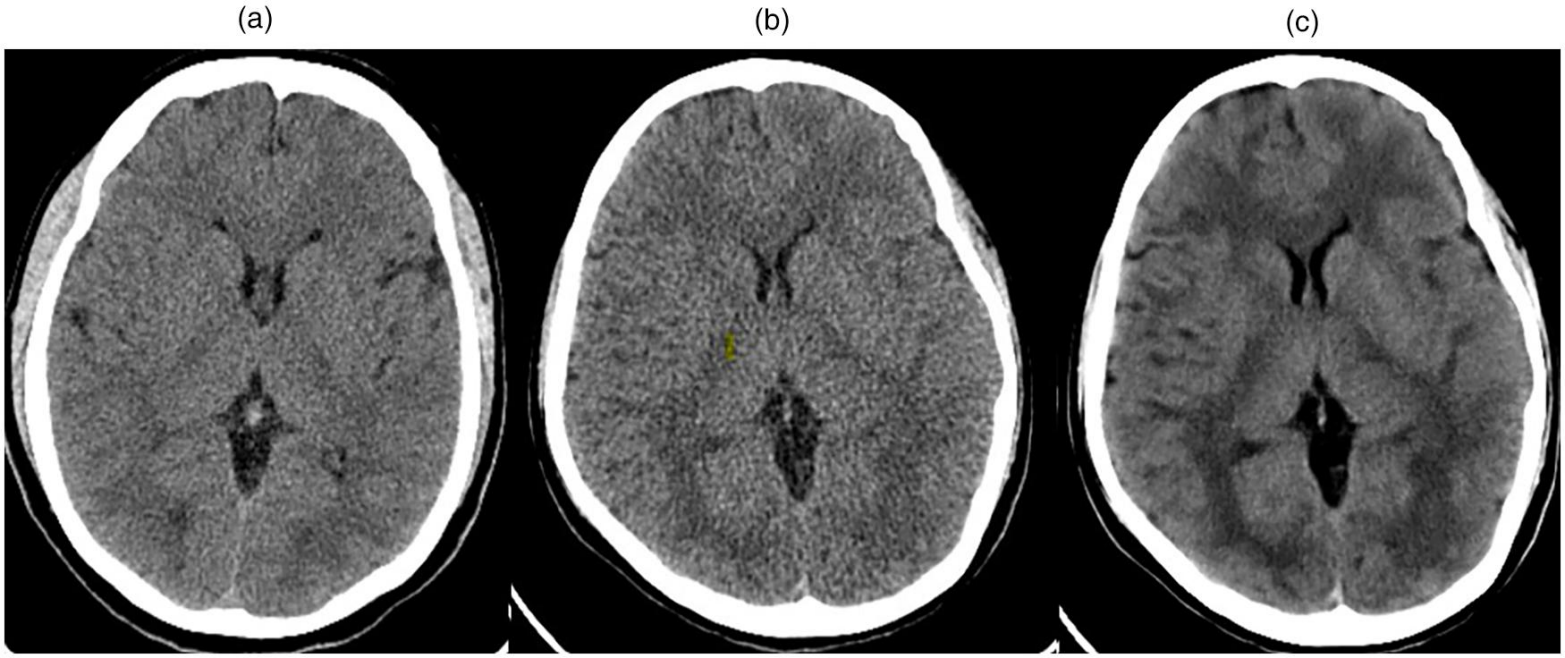
Axial CT image of a 58-year old man with **(a)** standard-dose-iDose^4^ and axial CT image of a 59-year-old man with **(c)** low-dose-DLIR showing better delineation of the gray and white matter compared with low-dose-iDose^4^ **(b)**.

For GWD, SD-iDose^4^ demonstrated median scores of 3 (IQR 3-3; R1), while LD-iDose^4^ showed reduced scores of 2.5 (IQR 2–3; R1), suggesting a decline in IQ with dose reduction; LD-DLIR showed the highest scores of 4.5 (IQR 4–5; R1), with statistically significant differences among the groups (*p* < 0.05). For OIQ, SD-iDose^4^ demonstrated median scores of 3–3.5, which decreased to 2.5–3 with LD-iDose^4^, whereas LD-DLIR demonstrated significantly higher scores of 4–4.5 (*p* < 0.05), suggesting improved IQ. For SIN, SD-iDose^4^ showed scores of 3–3.5, LD-iDose^4^ showed increased noise with lower scores of 2.5–3, and LD-DLIR significantly improved scores of 4–4.5, suggesting significant noise reduction (*p* < 0.05). However, no significant difference (*p* > 0.05) was noted in IA between the three groups. LD-DLIR significantly improved subjective IQ compared with SD-iDose^4^ and LD-iDose^4^ for NCCT head imaging, with excellent interobserver agreement (*k* = 0.892–0.979) (Table 4 and Figures 5a–c).

**Table 4:** Subjective IQ parameter comparison among standard-dose-iDose ^4^, low-dose-iDose^4^, and low-dose- DLIR.

Subjective image quality	Standard-dose-iDose^4^			Low-dose-iDose^4^			Low-dose-DLIR			*p*-Value SD-Dose^4^ vs. LD-iDose^4^ R1 and R2	*p*-Value SD-iDose^4^ vs. LD-DLIR R1 andR2	*p*-Value LD-iDose^4^ vs. LD-DLIR R1 andR2
	Reader 1 median (IQR)	Reader 2 median (IQR)	*k*-value	Reader 1 median (IQR)	Reader 2 median (IQR)	*k*-value	Reader 1 median (IQR)	Reader 2 median (IQR)	k-value			
Gray–white matter differentiation (GWD)	3 (3, 3)	3 (3, 3)	0.952	2.5 (2,3)	2.5 (2,3)	0.912	4.5 (4, 5)	4.5 (4,5)	0.979	<0.05	<0.05	<0.05
Overall image quality (OIQ)	3.5 (3, 4)	3 (3, 3)	0.892	3 (3,3)	2.5 (2,3)	0.924	4.5 (4, 5)	4 (4,5)	0.965	<0.05	<0.05	<0.05
Subjective image noise (SIN)	3.5 (3, 4)	3 (3, 4)	0.907	3 (3,3)	2.5 (2,3)	0.931	4.5 (4, 5)	4 (4,5)	0.908	<0.05	<0.05	<0.05
Image artifacts (IA)	3 (3, 4)	3 (3, 4)	0.919	3 (3, 4)	3 (3, 4)	0.892	3 (3, 4)	3 (3,4)	0.98	>0.05	>0.05	>0.05

SD, standard dose; LD, low dose; DLIR, deep learning image reconstruction.

#### 3.2.2 Objective IQ analysis

For IN at the BGL, SD-iDose^4^ demonstrated values of [GMT: median 4.80 (IQR: 4.15–5.39); WMPIC: 4.36 (3.76–4.90); R1], which increased with LD-iDose^4^ [6.25 (5.50–6.93); 5.32 (4.18–6.21); R1], indicating a decline in IQ with dose reduction. However, LD-DLIR showed significantly lower IN values [2.30 (2.11–2.44); 1.98 (1.59–2.50); R1] (*p* < 0.05). At the CSL, similar results were observed, with SD-iDose^4^ values of [ACGM: 4.14 (3.53–4.72); FWM: 3.90 (3.35–4.63)], increasing with LD-iDose^4^ [5.04 (4.28–5.85); 4.02 (3.38–5.06); R1], while LD-DLIR demonstrated reduced values [2.19 (1.84–2.63); 2.06 (1.64–2.47); R1] (*p* < 0.05). At the PF, IN increased from SD-iDose^4^ [6.05 (5.33–6.78); R1] to LD-iDose^4^ [6.93 (5.59–6.95)], whereas LD-DLIR showed significantly lower values [2.99 (2.56–3.66); R1] (*p* < 0.05).

For SNR at the BGL, SD-iDose^4^ demonstrated values of [GMT: 7.34 (6.37–8.32); WMPIC: 6.49 (5.94–7.62); R1], which decreased with LD-iDose^4^ [5.61 (5.03–6.39); 5.18 (4.57–6.49); R1], while LD-DLIR showed markedly higher values [16.29 (14.91–17.58); 14.75 (11.16–18.33); R1] (*p* < 0.05). At the CSL, SNR decreased from SD-iDose^4^ [ACGM: 8.19 (7.19–9.39); FWM: 7.38 (6.14–8.59); R1] to LD-iDose^4^ [6.59 (5.89–7.94); 6.95 (5.39–8.41); R1], whereas LD-DLIR demonstrated significantly higher values [16.27 (13.04–19.09); 14.00 (11.69–17.19); R1] (*p* < 0.05).

For CNR at the BGL, SD-iDose^4^ demonstrated values of [GMT–WMPIC: 0.98 (0.80–1.21); R1], which decreased with LD-iDose^4^ [0.82 (0.66–1.02); R1], while LD-DLIR showed significantly higher values [2.59 (2.29–2.93), R1] (*p* < 0.05). At the CSL, similar results were observed, with SD-iDose^4^ values of [ACGM–FWM: 0.93 (0.73–1.07); R1], decreasing with LD-iDose^4^ [0.85 (0.64–1.06); R1], and significantly increased with LD-DLIR [2.15 (1.58–3.12); R1] (*p* < 0.05). (Table 5)

**Table 5:** Objective IQ parameter comparison among standard-dose-iDose^4^, low-dose-iDose^4^, and low-dose-DLIR.

Region	Standard-dose-iDose^4^		ICC	Low-dose-iDose^4^		ICC	Low-dose-DLIR		ICC	*p*-Value SD- iDose^4^ vs. LD-iDose^4^ R1 and R2	*p*-Value SD- iDose^4^ vs. LD-DLIR R1 and R2	*p*-Value LD- iDose^4^ vs. LD-DLIR R1 and R2
	R1 (median and IQR)	R2 (median and IQR)		R1 (median and IQR)	R2 (median and IQR)		R1 (median and IQR)	R2 (median and IQR)				
Image noise												
BGL												
GMT	4.80 (4.15,5.39)	4.85 (4.15,5.48)	0.881	6.25 (5.50,6.93)	6.15 (5.49,6.84)	0.870	2.30 (2.11,2.44)	2.31 (2.12,2.47)	0.876	<0.05	<0.05	<0.05
WMPIC	4.36 (3.76,4.90)	4.38 (3.80,4.89)	0.926	5.32 (4.18,6.21)	5.19 (4.15,6.18)	0.856	1.98 (1.59,2.50)	2.21 (1.63,2.65)	0.814	<0.05	<0.05	<0.05
CSL												
ACGM	4.14 (3.53,4.72)	4.22 (3.60,4.70)	0.819	5.04 (4.28,5.85)	5.08 (4.27,5.76)	0.896	2.19 (1.84,2.63)	2.17 (1.75,2.60)	0.872	<0.05	<0.05	<0.05
FWM	3.90 (3.35,4.63)	3.96 (3.31,4.62)	0.853	4.02 (3.38,5.06)	4.88 (3.38,4.88)	0.901	2.06 (1.64,2.47)	2.02 (1.58,2.46)	0.810	<0.05	<0.05	<0.05
Posterior fossa index												
PF IN	6.05 (5.33,6.78)	5.98 (5.31, 6.72)	0.847	6.93 (5.59,6.95)	7.13 (5.59, 7.23)	0.912	2.99 (2.56,3.66)	3.05 (2.60,3.75)	0.812	<0.05	<0.05	<0.05
Signal-to-noise ratio												
BGL												
GMT	7.34 (6.37,8.32)	7.32 (6.27,8.26)	0.885	5.61 (5.03, 6.39)	5.75 (5.05,6.35)	0.882	16.29 (14.91,17.58)	16.36 (14.88,17.57)	0.844	<0.05	<0.05	<0.05
WMPIC	6.49 (5.94,7.62)	6.51 (6.04,7.43)	0.825	5.18 (4.57,6.49)	5.26 (4.59,6.52)	0.875	14.75 (11.16,18.33)	14.69 (10.95,18.09)	0.821	<0.05	<0.05	<0.05
CSL												
ACGM	8.19 (7.19,9.39)	8.14 (7.42,9.65)	0.812	6.59 (5.89,7.94)	6.51 (5.96,7.97)	0.901	16.27 (13.04,19.09)	16.34 (13.50,20.67)	0.861	<0.05	<0.05	<0.05
FWM	7.38 (6.14,8.59)	7.39 (6.05,8.64)	0.836	6.95 (5.39, 8.41)	6.97 (5.47,8.41)	0.956	14.00 (11.69,17.19)	14.21 (11.76,17.68)	0.846	<0.05	<0.05	<0.05
Contrast-to-noise ratio												
BGL												
GMT WMPIC	0.98 (0.80,1.21)	0.99 (0.83,1.20)	0.91	0.82 (0.66, 1.02)	0.83 (0.67,1.03)	0.956	2.59 (2.29,2.93)	2.61 (2.29,2.91)	0.889	<0.05	<0.05	<0.05
CSL												
ACGM-FWM	0.93 (0.73,1.07)	0.95 (0.77, 1.09)	0.867	0.85 (0.64, 1.06)	0.86 (0.65,1.07)	0.972	2.15 (1.58,3.12)	2.29 (1.64,3.18)	0.828	<0.05	<0.05	<0.05

BGL, basal ganglia level; GMT, gray matter thalamus; WMPIC, white matter posterior limb of internal capsule; CSL, centrum semiovale level; ACGM, adjacent cortical gray matter; FWM, frontal white matter; PF-IN, posterior fossa index; IQR, interquartile range; R1, reader 1; R2, reader 2; SD, standard dose; LD, low dose.

The ICC used to assess interobserver agreement showed good-to-excellent agreement between the readers.

#### 3.2.3 Radiation dose metrics

Compared with the SD NCCT head protocol, the LD protocol demonstrated a statistically significant difference (*p* < 0.05) and reductions of 58.42% in CTDIvol (19.28 ± 0.02 vs. 46.37 ± 0.03 mGy), 59.20% in DLP (479.03 ± 49.74 vs. 1174.37 ± 54.41 mGy/cm), 58.32% in SSDE (18.59 ± 2.25 vs. 44.61 ± 4.05 mGy), and 59.11% in ED (1.01 ± 0.10 vs. 2.47 ± 0.32) (Table 6).

**Table 6:** Comparison of RD between groups A and B.

Radiation dose (RD) indices	Group A mean ± SD	Group B mean ± SD	Dose reduction (%)	*p*-Value
CTDIvol (mGy)	46.37 ± 0.03	19.28 ± 0.02	58.42	<0.05
DLP (mGy/cm)	1,174.37 ± 54.41	479.03 ± 49.74	59.20	<0.05
SSDE (mGy)	44.61 ± 4.05	18.59 ± 2.25	58.32	<0.05
Effective dose (mSv)	2.47 ± 0.32	1.01 ± 0.06	59.10	<0.05

#### 3.2.4 Lesion conspicuity

**Figure 6:**
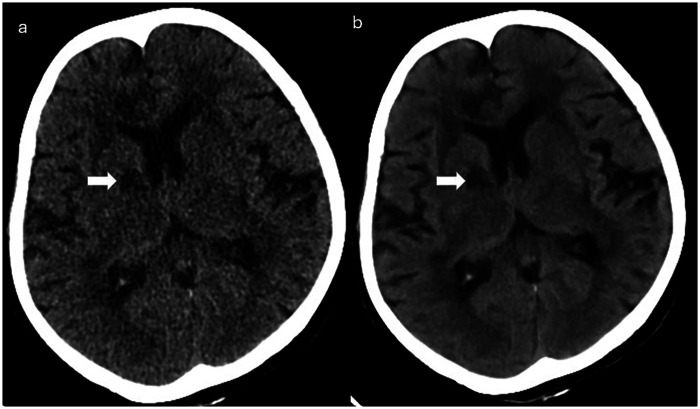
Axial CT LD images of an 80-year-old woman with a right lentiform nucleus acute infarct. The small hypoattenuation area (arrows) was clearly delineated in DLIR **(b)** while preserving image noise compared with iDose^4^ **(a)**.

**Figure 7:**
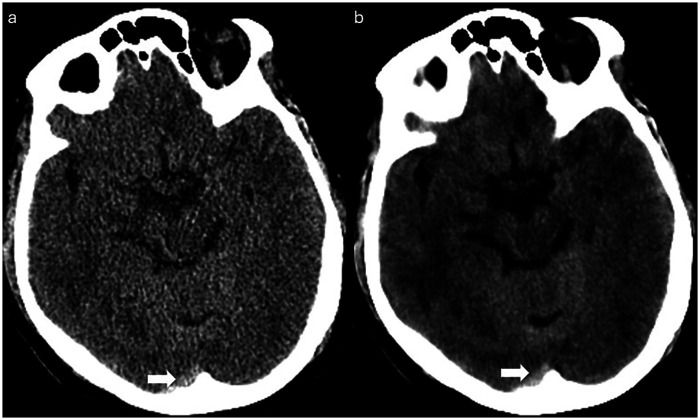
Axial CT LD images of a 56-year-old woman with right occipital acute extradural hemorrhage. The small hyperattenuation area was clearly delineated in DLIR **(b)** while preserving image noise compared with iDose^4^ **(a)**.

**Figure 8:**
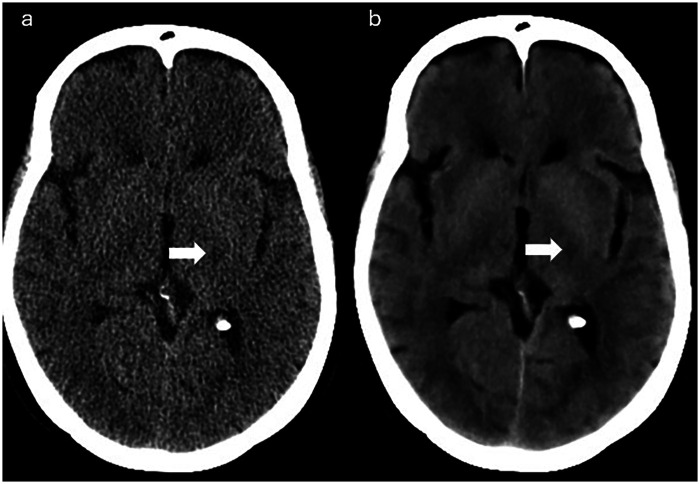
Axial CT LD images of a 36-year-old woman with a left chronic infarction. The small hypoattenuation area (arrows) was clearly delineated in DLIR **(b)** while preserving image noise compared with iDose^4^ **(a)**.

A total of 39 lesions were identified in 39 patients on low-dose images and were subjected to conspicuity assessment. These included cerebrovascular diseases, including acute ischemia, subdural hemorrhage, intracranial hemorrhage, postoperative changes, and intracranial tumors (meningioma, glioblastoma multiforme). All these lesions were visible on all image reconstructions. The mean conspicuity scores demonstrated a clear improvement with LD-DLIR compared with LD-iDose^4^ for both readers. For Reader 1, the mean score increased from 1.05 ± 0.22 with LD-iDose^4^ to 2.00 ± 0.00 with LD-DLIR, while for Reader 2, the mean score increased from 1.02 ± 0.16 to 2.00 ± 0.00 (Table 7). For LD-iDose^4^, both readers assigned score 1 (Reader 1: 39/39, 100%; Reader 2: 38/39, 97.4%), with only one case receiving a score of 2 by Reader 2 (2.5%). However, in the LD-DLIR group, all cases were assigned a score of 2 by both readers (Reader 1: 39/39, 100%; Reader 2: 39/39, 100%), with no cases receiving Score 1. The percentage of scores (1 and 2) for the reconstruction levels is provided in Table 8 and Figures 6–8.

**Table 7:** Mean and SD conspicuity scores between LD-iDose^4^ and LD-DLIR.

Group	LD-iDose^4^		LD-DLIR		*p*-Value
	Reader 1	Reader 2	Reader 1	Reader 2	
Mean (SD)	1.05 (0.22)	1.02 (0.16)	2 (0)	2 (0)	<0.05

**Table 8:** Number and percentage of score 1 (lesion was least conspicuous among the two-image series) and score 2 (lesion was most conspicuous among the two-image series) assigned for each image by each reader.

Group	Score 1		Score 2	
	Reader 1	Reader 2	Reader 1	Reader 2
LD-iDose^4^	39 (100%)	38 (97.4%)	0 (0%)	1 (2.5%)
LD-DLIR	0 (0%)	0 (0%)	39 (100%)	39 (100%)

## 4 Discussion

Our study evaluated IQ between LD-DLIR (Precise Image), LD-iDose^4^, and SD-iDose^4^ and also further evaluated radiation dose reduction between SD-iDose^4^ and LD-DLIR for NCCT head imaging. We also assessed the lesion conspicuity between the LD-IR and LD-DLIR groups. The results demonstrated that LD-DLIR (Precise Image) showed better subjective and objective IQ values than LD-iDose^4^ and SD-iDose^4^, while providing a significant reduction in radiation dose for NCCT head imaging. In addition, LD-DLIR showed greater lesion conspicuity than LD-iDose^4^. These findings suggest that simultaneous reduction of kVp and mAs in non-contrast CT head imaging with DLIR can substantially lower the RD while improving IQ and enhancing lesion conspicuity.

### 4.1 Image noise

Our study reported a greater reduction in IN (47%–55%) compared with the study by Nagayama et al. (22), which reported a reduction of 15–17.6% on LD-DLIR NCCT brain imaging compared with SD- hybrid iterative reconstruction (HIR). Nagayama et al. (22) also compared SD-HIR with several LD reconstruction techniques [LD-HIR, LD-model-based iterative reconstruction (MBIR), LD-DLIR] and reported that LD-DLIR reduced IN compared with SD-HIR and LD-HIR at a 25% reduction in ED. In the present study, LD-DLIR reduced IN by 55%–65% compared with LD-iDose^4^, whereas Nagayama et al. (22) reported a more modest reduction of 20%–25% compared with LD-DLIR over LD-HIR and LD-MBIR. Pula et al. (23) retrospectively compared head CT images obtained with DLIR and the adaptive statistical iterative reconstruction technique (ASIR-V) and reported that DLIR significantly reduced IN, with improvements in SNR and CNR.

DLIR also demonstrated lower median IN compared with adaptive iterative dose reduction (AIDR-3D; median 4.7 vs. 5.3; 19.6% IN reduction). NPS analysis showed that DLIR shifted noise to lower average frequency, while AIDR-3D broadened the noise pattern (24). Furthermore, DLIR significantly reduced IN compared with HIR and improved the conspicuity of acute infarct, especially within 24 h (25). Two additional studies reported a progressive reduction in IN as DLIR strength increased (26, 27). (Table 9)

**Table 9:** Exposure parameters (kVp and mAs), reconstruction levels, key findings, and radiation dose reduction between the present study and others.

Author (year)	This study	Nagayama et al. (22)	Pula et al. (23)	Cozzi et al. (24)	Okimoto et al. (25)	Kim et al. (26)	Alagic et al. (27)	Oosteveen et al. (28)
kVp	100 kvp (low dose) 120 kVp (standard dose)	120 kVp (standard and low doses)	120 kVp	120 kVp	120 kVp	120 kVp	120 kVp	120
mAs	200 mAs (low dose) 290 mAs (standard dose)	350 mA (standard dose) 280 mA (low dose)	-	125 mAs	Automatic tube current modulation	100–300 mA	290 mA	350–360 mAs, 140/225 mAs
Reconstruction levels	Precise Image vs. iDose^4^	AiCE vs. HIR vs. MBIR	True fidelity vs. ASIR-V	AiCE vs. AIDR-3D	AiCE vs. AIDR-3D	True fidelity vs. ASIR-V	True fidelity vs. ASIR-V	AiCE vs. hybrid IR vs. MBIR
Key findings with respect to IQ	47–55% reduction in image noise with DLIR LD-DLIR reduced IN by 55–65% compared with LD-idose^4^ 90%–127% improvement in SNR 131–164% improvement in CNR	15–17.6% reduction in image noise with DLR IN reduction of 20–25% with LD-DLIR compare with LD-HIR and LD-MBIR 35–37.5% increase in CNR	145% and 160% increase in SNR in supra- and infratentorial regions CNR showed an increase of 171.5% in the supratentorial region and a 59.3% increase in the infratentorial region	19.6% (median) reduction in IN CNR improved to 39% at the cortical level and 65% at the thalamic level	DLIR reduced IN by 12.4% (lateral ventricle, 26.6% (putamen), 34.0% (white matter) CNR increased to 116.7% Lesion white matter contrast increased to 36.4%	IN was reduced with increase in DLIR strength (23.6–51.1%) CNR was improved at all DLIR levels	DLIR-H and M lowered image noise SNR was increased by up to 82.9% CNR increased by up to 53.3%	DLIR demonstrated lower IN compared with hybrid-IR and MBIR, with reductions of 12.5% and 9.7%, respectively DLIR showed improved SDNRs of 26.3% and 20% compared with MBIR
% Reduction in radiation dose	59%	25%	44% reduction in CTDI vol	-	-	-	-	-

MBIR, model-based iterative reconstruction; HIR, hybrid iterative reconstruction; SNR, signal-to-noise ratio; CNR, contrast-to-noise ratio; AiCE, advanced intelligent clear-IQ engine; IN, image noise.

### 4.2 Gray–white matter differentiation, SNR, and CNR

DLIR was consistently associated with enhanced GWD, improved noise texture, and enhanced image sharpness while maintaining overall diagnostic acceptability (22–28). Our study also demonstrated enhanced GWD, improved OIQ, and reduced SIN with LD DLIR compared with SD-iDose^4^ and LD-iDose^4^. The current study demonstrated a marked improvement in GWD, with subjective scores increasing by 80% with LD-DLIR compared with LD-iDose^4^. This finding was consistent with Nagayama et al. (22), who reported improved GM-WM contrast by 25% at the BGL level and 14% in the posterior fossa with LD-DLIR compared with LD-HIR and LD-MBIR. The ability of the DLIR to preserve anatomical detail at LDs, particularly in the basal ganglia and posterior fossa, has been reported in previous studies (22, 23). Improvements in structural delineation were also noted in head trauma imaging, where higher strengths of DLIR eliminated non-diagnostic cases during intracranial hemorrhage evaluation (27). Studies focusing on acute ischemia (23, 25) demonstrated that DLIR improved the visualization of subtle artifacts in LD protocols compared with IR.

With respect to SNR and CNR, DLIR showed increases in SNR of up to 145% in the supratentorial region and 160% in the infratentorial region compared with ASIR-V. Similarly, CNR also increased by 171.5% in the supratentorial region and 59.3% in the infratentorial region (23). LD-DLIR and LD-MBIR showed improved GM and WM differentiation, along with higher CNR, compared with SD-HIR. Thus, DLIR maintains or increases CNR while preserving noise texture at lower doses (22).

Our study also reported higher SNR and CNR values with DLIR at reduced doses compared with the findings reported by Nagayama et al. (22) (Table 8). A substantial increase in SNR and CNR of 150%–200% was noted in the present study with LD-DLIR compared with LD-iDose^4^, similarly to the study by Pula et al. (23). Higher CNR was noted with DLIR at cortical (2.5 vs. 1.8) and thalamic (2.8 vs. 1.7) levels (24). In addition, CNR improved with an increase in DLIR levels at CSL and BGL levels (28). DLIR has also been shown to increase SNR and CNR by up to 82.9% and 53.3%, respectively, compared with ASIR-V (27).

### 4.3 Radiation dose

Our study noted higher radiation dose reductions of 58.4% CTDIvol and 59.2% DLP with the LD protocol, compared with previous studies that reported 25% CTDIvol and 44% CTDIvol, along with 15% DLP reduction (22, 23).

### 4.5 Lesion conspicuity

Okimoto et al. (28) evaluated lesion conspicuity using a five-point visual scoring system across vascular territories to assess acute infarct depiction and reported that DLIR improves early infarct detection. Nagayama et al. (22) evaluated lesion conspicuity using a three-point ranking scale and compared three reconstruction algorithms, demonstrating superior lesion visibility with DLIR. Our study also reported superior lesion detection with LD-DLIR compared with LD-IR.

### 4.6 Limitations

This study has a few limitations. First, the study focused on a vendor-specific DLIR technique; thus, the applicability of these findings to other DLIR algorithms remains uncertain. Second, the study was conducted at a single institution using its own specific examination protocols; further multicenter research incorporating a variety of protocols is warranted.

Our study concluded that LD non-contrast CT head imaging reconstructed with DLIR offers superior noise reduction, enhanced SNR and CNR, and lesion conspicuity, compared with the SD and LD iterative reconstruction methods (iDose^4^). These results demonstrate that using DLIR in the clinical protocol supports radiation exposure without compromising diagnostic image quality, thereby promoting safer and more efficient neuroimaging practice.

## Data Availability

The raw data supporting the conclusions of this article will be made available by the authors, without undue reservation.
